# Technological Innovations and Data-Driven Support for Older Adults

**DOI:** 10.2196/48547

**Published:** 2023-05-10

**Authors:** Yun Jiang, Jinjiao Wang

**Affiliations:** 1 School of Nursing University of Michigan Ann Arbor, MI United States; 2 School of Nursing University of Rochester Rochesteer, MI United States

**Keywords:** technological innovation, JMIR Aging, older adults, innovation, technology, digital ageism, digital health, older adult care

## Abstract

Entering a new digital era where novel devices and emerging technologies, including artificial intelligence, are playing an incredible role with significant impact on health and health care delivery, *JMIR Aging* commits to supporting the community of patients and families, clinicians, and scientists to improve the efficiency, equity, and effectiveness of older adult care through the dissemination of cutting-edge evidence.

One in every six people in the world will be aged 60 years or over in 2030, totaling 2.1 billion by 2050 [1]. As people age, they are more likely to experience complex health conditions that will need support from families, health systems, and society. To “add life to years” of older adults, one promising solution may lie in the appropriate use of technology. To date, technological innovations in health care, such as smart wearable devices [2], remoting patient monitoring [3], socially assistive robots [4], augmented reality and virtual reality [5], automatic medication dispensers [6], and synchronized electronic health record systems that enable patients to virtually communicate with the care team [7], have been shown to help older adults stay physically active, live independently, monitor changes in medical conditions, and build social connections. More recently, advanced data science approaches have also been used to provide clinicians with point-of-care support for informed patient care decisions [8], speed up vaccine development [9], and support patients with shared decision-making [10].

Though the rise of technological and data science innovations brings promises, it also poses challenges. For example, many older adults, due to the lack of familiarity with technology, digital literacy, access to technological tools, or access to internet services, are negatively affected by the digital divide [11,12]. Digital ageism—the neglect of older adults’ needs, experience, and preferences in the user interaction design of some of the technologies—makes it even more difficult for them to engage in and enjoy the use of digital health technologies.

As an open-access journal, *JMIR Aging* strives to serve as a platform to support information-sharing and communications about older adults’ health and health care among clinicians, patients, caregivers, researchers, and policy makers. Our mission is to promote the use of technological innovations and data science to inform and improve health care services and health outcomes for older adults. Our focus includes digital health; emerging technologies; health informatics applications; patient education; and preventative care, clinical care, home care, and self-management support for older adults. *JMIR Aging* also covers aging-focused big data analytics using data from electronic health record systems, health insurance databases, federal reimbursement databases (eg, US Medicare and Medicaid), and other large data sets.

Founded in 2018, *JMIR Aging* has published 254 articles on various topics, such as the use of remote monitoring and artificial intelligence or robotic-driven systems [13], telehealth visits among underserved older adult populations [14], and fall risk prevention mobile health solutions [15]. *JMIR Aging* has been indexed in PubMed, PubMed Central, DOAJ, Scopus, and the Emerging Sources Citation Index (Clarivate), and it is expected to receive an influential impact factor in 2023.

Entering a new digital era where novel devices and emerging technologies, including artificial intelligence, are playing an incredible role with significant impact on health and health care delivery, *JMIR Aging* commits to supporting the community of patients and families, clinicians, and scientists to improve the efficiency, equity, and effectiveness of older adult care through the dissemination of cutting-edge evidence.
